# Genome-wide association study of nausea and vomiting during pregnancy in Japan: the TMM BirThree Cohort Study

**DOI:** 10.1186/s12884-024-06376-4

**Published:** 2024-03-20

**Authors:** Yudai Yonezawa, Ippei Takahashi, Hisashi Ohseto, Fumihiko Ueno, Tomomi Onuma, Aoi Noda, Keiko Murakami, Mami Ishikuro, Taku Obara, Shinichi Kuriyama

**Affiliations:** 1 Division of Molecular Epidemiology, Tohoku University Graduate School of Medicine, 2-1 Seiryo-machi, Aoba-ku, Sendai, Miyagi 980-8575 Japan; 2 Innovation Division, KAGOME CO., LTD, 17 Nishitomiyama, Nasushiobara, Tochigi 329- 2762 Japan; 3 Tohoku Medical Megabank Organization, Tohoku University, 2-1 Seiryo-machi, Aoba-ku, Sendai, Miyagi 980-8573 Japan; 4 Department of Pharmaceutical Sciences, Tohoku University Hospital, 1-1 Seiryo-machi, Aoba- ku, Sendai, Miyagi 980-0872 Japan; 5 International Research Institute of Disaster Science, Tohoku University, 468-1 Aramakiaoba, Aoba-ku, Sendai, Miyagi 980-8572 Japan

**Keywords:** Hyperemesis gravidarum, Nausea and vomiting of pregnancy, GWAS, Genetic, GDF15, Japan, PGPEP1, TRPC6

## Abstract

**Background:**

Nausea and vomiting during pregnancy (NVP) and hyperemesis gravidarum (HG), common conditions affecting most pregnant women, are highly heritable and associated with maternal and fetal morbidity. However, the pathologies underlying NVP and HG and their associated loci are scarce.

**Methods:**

We performed genome-wide association studies (GWAS) of NVP in pregnant women (*n* = 23,040) who participated in the Tohoku Medical Megabank Project Birth and Three-Generation Cohort Study in Japan from July 2013 to March 2017. Participants were divided into discovery (*n* = 9,464) and replication (*n* = 10,051) stages based on the platform used for their genotyping. Loci that achieved the genome-wide significance level (*p* < 5.0 × 10^− 8^) in the discovery stage were selected for genotyping in the replication stage. A meta-analysis integrating the discovery and replication stage results (*n* = 19,515) was conducted. NVP-related variables were identified as categorical or continuous.

**Results:**

GWAS analysis in the discovery phase revealed loci linked to NVP in two gene regions, 11q22.1 (rs77775955) and 19p13.11 (rs749451 and rs28568614). Loci in these two gene regions have also been shown to be associated with HG in a White European population, indicating the generalizability of the GWAS analyses conducted in this study. Of these, only rs749451 and rs28568614 at 19p13.11 reached the genome-wide suggestive level (*p* < 1.0 × 10^− 5^) in the replication stage; however, both loci were significant in the meta-analysis.

**Conclusions:**

NVP-related loci were identified in the Japanese population at 11q22.1 and 19p13.11, as reported in previous GWAS. This study contributes new evidence on the generalizability of previous GWAS on the association between genetic background and NVP.

**Supplementary Information:**

The online version contains supplementary material available at 10.1186/s12884-024-06376-4.

## Background

Nausea and vomiting of pregnancy (NVP) is a common disorder affecting approximately 80% of all pregnant women [1]. Severe nausea and vomiting, known as hyperemesis gravidarum (HG), occurs in 0.3–3.6% of pregnant women worldwide [2]. HG is associated with maternal and fetal morbidity and has been linked to nutritional deficiencies during pregnancy, oesophageal damage, mental health effects, and birth outcomes such as preterm delivery and low birth weight [3]. Therefore, it is important to elucidate the pathologies of NVP and HG, which have serious implications for maternal and fetal health.

In a previous study on twins, the heritability of NVP was estimated to be approximately 73% for presence and 53% for severity [4], suggesting that elucidating the genetic mechanism is essential to gain insights into the disease’s etiology. Genome-wide association studies (GWAS) have gained interest in genetics research to identify genetic variations underlying particular diseases. However, to our knowledge, only three previous GWAS have identified such an association in patients with HG or excessive vomiting in pregnancy. Fejzo et al. identified the association between loci related to *GDF15* and *IGFBP7* (placentation, appetite, and cachexia genes) and HG in European ancestry populations [5]. Changalidis et al. also identified the association between *GDF15*-related loci and excessive vomiting in pregnancy in European ancestry populations [6]. Furthermore, the GWAS meta-analysis of European and Asian ancestry populations, including British Bangladeshis and Pakistanis, revealed the association between the *GDF15*-related locus and HG [7]. A recent study reported that the severity of nausea and vomiting during pregnancy is caused by the interaction between fetal-derived GDF15 and maternal sensitivity to this peptide [8]. However, no such studies involving East Asian ancestry populations, including Japanese populations, have been reported. Furthermore, allele frequency (AF) and linkage disequilibrium (LD) differ between East Asian and other ancestry populations. Therefore, we speculated that GWAS for NVP and HG in the Japanese population may identify unique loci and provide evidence for the generalizability of GWAS results with other ancestries. Moreover, we hypothesized that the analysis of different classifications of NVP severity based on the presence or absence of vomiting and appetite could identify the new genetic factors related to NVP severity.

To test these speculations, the present study aimed to clarify the association between genetic factors and NVP in Japanese participants of the Tohoku Medical Megabank Project Birth and Three Generations Cohort Study (TMM BirThree Cohort Study). The participants were divided into discovery and replication stages for a two-step evaluation. The key loci underlying NVP and HG were analyzed using a meta-analysis conducted by combining the results from the discovery and replication stages. The present study will provide new evidence on the association between genetic background and NVP, which may contribute to understanding the pathologies of NVP and HG.

## Methods

### Study design and population

**Fig. 1:**
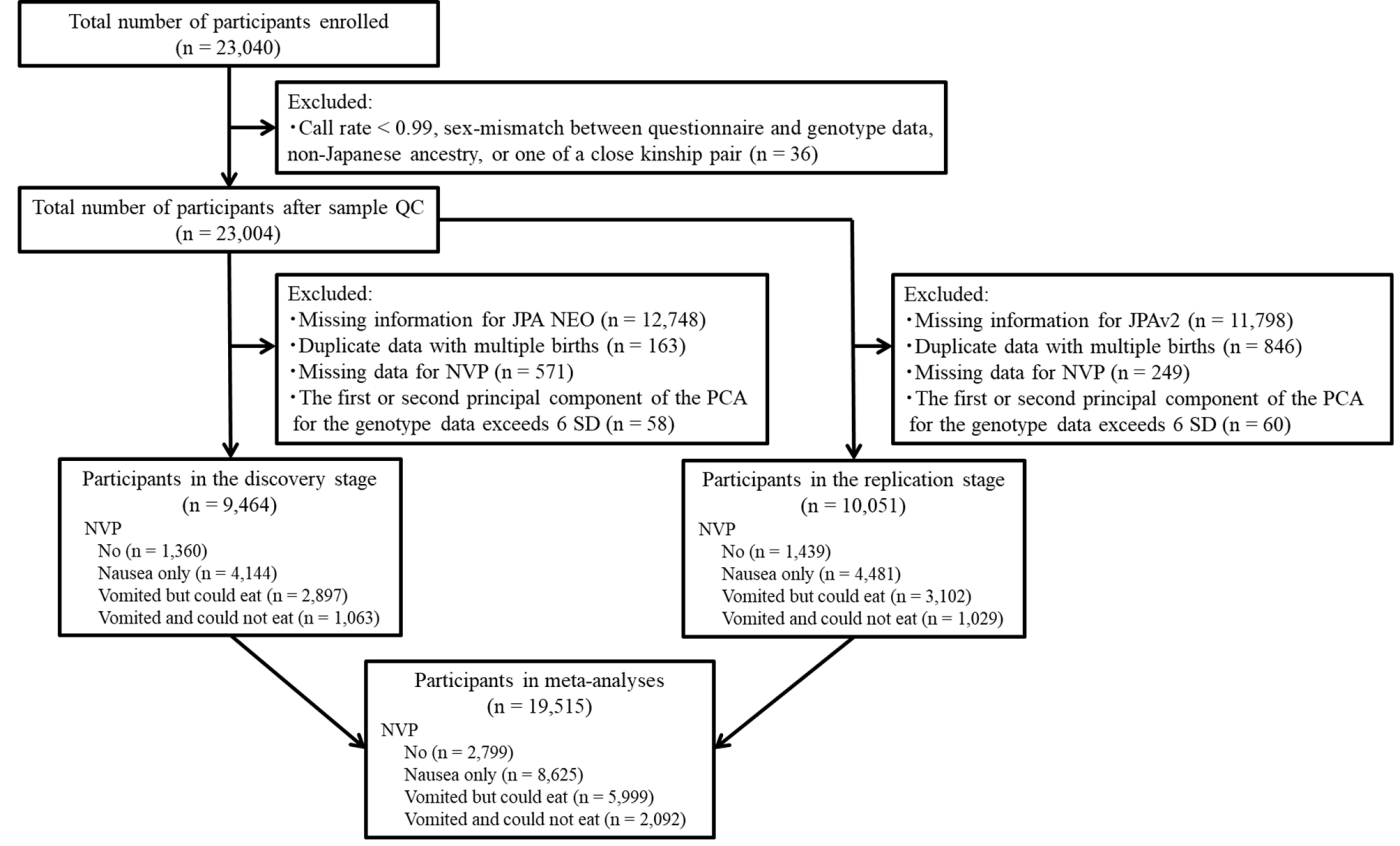
Flow chart of the participants in this study. A total of 23,040 pregnant women were recruited in this study from July 2013 to March 2017. After excluding the participants based on the shown exclusion criteria, 9,464 and 10,051 participants were included in the discovery and replication stages, respectively. The participants in the discovery and replication stages were combined in the meta-analysis stage. JPA NEO, Axiom Japonica Array NEO; JPAv2, Axiom Japonica Array v2; NVP, nausea and vomiting of pregnancy; QC, quality control

Participants included in this study were pregnant women enrolled in the Tohoku Medical Megabank Project Birth and Three-Generation Cohort Study (TMM BirThree Cohort Study) in Japan reported previously [9, 10]. A total of 23,040 pregnant women were recruited in this study from July 2013 to March 2017. In the TMM BirThree Cohort Study, genotyping was started using JPav2 from families for whom biological samples were available and gradually switched to genotyping with JPA NEO as the study progressed. Thus, participants included during the early phases of the study were more frequently genotyped with Axiom Japonica Array v2 (JPav2), while those in the late phases of the study were more frequently genotyped with Axiom Japonica Array NEO (JPA NEO). Participants compatible with the following criteria were excluded from the analysis: call rates < 0.99, sex mismatch between questionnaire and genotype data, non-Japanese ancestry, or one of a pair of close relatives (PI_HAT > 0.1875). The participants were assigned to discovery and replication stages for a two-stage evaluation based on their genotyping information—those genotyped using JPA NEO (*n* = 10,256) were assigned to the discovery cohort, and those genotyped using JPAv2 (*n* = 11,206) were assigned to the replication stage. Finally, after excluding the participants using the criteria shown in Fig. 1, a total of 9,464 and 10,051 participants were included in the discovery and replication stages, respectively. Subsequently, all participants in these two groups (*n* = 19,515) were included in the meta-analysis. None of the participants were common between the discovery or replication stages.

### Genotyping, imputation, and QC

JPA NEO and JPav2 were used for genotyping the participants to create the imputed genomic dataset. JPA NEO was developed by modifying JPAv2, and the differences between the two were described in a previous study [11]. Variants that corresponded to any one of the following criteria: call rates < 0.95, minor allele frequency (MAF) < 0.01, and *p*-values for the Hardy–Weinberg equilibrium (HWE) test < 1.0 × 10^− 5^ were removed. An imputed genotype dataset was generated by pre-phasing using SHAPEIT2 [12] with the duoHMM option [13], which utilizes information on relatedness between individuals to enhance phasing accuracy. The phased genotypes were then imputed using a cross-imputed panel of 3.5KJPNv2 [14] and 1KGP3 [15] using IMPUTE4 software [16]. The ‘--merge_ref_panels’ option in IMPUTE2 [17] was used to construct a cross-imputation panel for 3.5KJPNv2 and 1KGP3. This resulted in an imputed genotype dataset in Oxford BGEN format (https://www.well.ox.ac.uk/gav/qctool/). The details of the imputation methods have been described previously [16, 17]. After imputation, the criteria for QC in the GWAS were as follows: call rate < 0.99, MAF < 0.01, *p*-values for the HWE test < 1.0 × 10^− 6^, and INFO score < 0.4. Finally, a total of 11,813,083 and 11,799,399 SNPs were included in the GWAS in the discovery and replication stages, respectively.

### Genome-wide association study (GWAS)

To verify the association between all eligible variants and the presence of NVP, a linear mixed-model analysis was performed using Genome-wide Complex Trait Analysis [18]. Associations between variants and binary variables were evaluated using a fast GLMM-based Genome-Wide Association tool (fastGWA-GLMM) to calculate regression coefficients, standard errors (SEs), and *p*-values [19]. The association between variants and continuous variables was assessed using the fast MLM-based Genome-Wide Association tool (fastGWA) to calculate regression coefficients, SEs, and *p*-values [20]. A sparse GRM was created with a cut-off value of 0.05 using the ‘--make-bK-sparse’ option and specified with the ‘--grm-sparse’ option in the GWAS analysis to adjust for genetic relatedness. Age and the first 10 principal components from principal component analysis (PCA) of the genotype data were used as adjustment variables. PCA was performed on the pre-imputed genomic data using the ‘--pca approx’ option in PLINK 2.0. Variants with *p* < 5.0 × 10^− 8^ and *p* < 1.0 × 10^− 5^ were considered genome-wide significant and suggestive, respectively. Variants with *p* < 5.0 × 10^− 8^ in the discovery stage were extracted and analyzed in the replication stage. For the replication stage, *p* < 1.0 × 10^− 5^ was used as the significance threshold. Loci that satisfied these two significance levels were considered replicated. Meta-analyses combining the discovery- and replication-stage results were performed using METAL [21] to calculate *p*-values and directions. Secondary GWAS analyses were performed using the data obtained from replication stage participants. LocusZoom 1.4 was used to generate regional association plots of SNPs [22]. The reference sequence from the 1000 Genomes Project for hg19 in Asians in November 2014 was used to calculate the LD in LocusZoom [15].

### Outcomes

The data on the NVP variable was collected from the participants using a questionnaire comprising a single question (Have you had any symptoms of morning sickness between this pregnancy and now?) with four answers (“no,” “nausea only,” “vomited but could eat,” and “vomited and could not eat”) during their early stages of pregnancy. Subsequently, the following four patterns were created using the NVP variable: binary 1 (“no” vs. “nausea only,” “vomited but could eat,” and “vomited and could not eat”), binary 2 (“no” and “nausea only” vs. “vomited but could eat” and “vomited and could not eat”), binary 3 (“no,” “nausea only,” and “vomited but could eat” vs. “vomited and could not eat”), binary 4 (“no” vs. “vomited and could not eat”), and continuous (regarding four categories as consecutive severity grading). Binary 1 was divided according to whether the person had nausea, binary 2 according to whether the person had vomiting, and binary 3 according to whether the person could eat food. Binary 4 was created to compare the group with no symptoms of NVP with the group with severe symptoms.

### Statistical analysis

Basic characteristics are presented as the means and standard deviations (SDs) for continuous variables and as frequencies and percentages for categorical variables in the discovery and replication cohorts. In addition to age and the NVP variable, other characteristics, such as height (cm), pre-pregnancy weight (kg), gestational age at acquisition of the NVP variable (weeks), parity (never, one or more), infant sex (male, female), gestational age at delivery (< 37 weeks, 37–41 weeks, ≥ 42 weeks), hypertensive disorder in pregnancy (HDP; yes, no) and gestational diabetes mellitus (GDM; yes, no) of the participants were also evaluated. Information on height and weight was collected using the questionnaire during early pregnancy. Information on parity, infant sex, gestational age at delivery, HDP and GDM was collected from medical records. All statistical analyses were performed using R (version 4.1.2). Functional annotation was performed using ANNOVAR software (2022Mar30; http://annovar.openbioinformatics.org/en/latest/). Functional analysis using HaploReg v4.2 was performed on lead SNPs where significant associations were identified [23]. The LD threshold for HaploReg was set to r^2^ = 0.2, and the population used for LD calculations was the 1000 Genomes Project Phase 1 Asian descent [15]. The GTEx portal was used to examine loci that satisfied genome-wide levels in the discovery or replication stages of the study and to identify expression quantitative trait loci (eQTL) in the database. Data from the genotype-tissue expression (GTEx) portal (https://gtexportal.org/home/; accessed on 3 October 2023) were used for the analyses.

## Results

### Characteristics of the study population

The participant characteristics for the discovery (*n* = 9,464) and replication (*n* = 10,051) stages are listed in Table 1. The participants in both groups had similar distributions for each variable, with an average age of 31.2 years. Approximately 15% of the pregnant women had no symptoms of NVP, whereas approximately 10% had severe NVP with vomiting and could not eat. The difference in gestational age at the acquisition of the NVP variable between the two stages was approximately 1.7 weeks.

**Table 1:** Characteristics of pregnant women included in this study and their infants

	Discovery stage (JPA NEO)	Replication stage (JPAv2)
n	9464	10,051
Age (years)	31.2 ± 5.0	31.2 ± 5.0
Height (cm)	158.4 ± 5.4	158.4 ± 5.3
Pre-pregnancy weight (kg)	53.5 ± 9.0	54.0 ± 9.2
Gestational age at acquisition of the NVP variable (weeks)	21.3 ± 9.7	19.6 ± 8.5
NVP		
No	1360 (14.4)	1439 (14.3)
Nausea only	4144 (43.8)	4481 (44.6)
Vomited but could eat	2897 (30.6)	3102 (30.9)
Vomited and could not eat	1063 (11.2)	1029 (10.2)
Parity		
Never	4311 (45.6)	5166 (51.4)
One or more	5142 (54.3)	4861 (48.4)
Missing	11 (0.1)	24 (0.2)
Infant sex		
Male	4827 (51.0)	5200 (51.7)
Female	4558 (48.2)	4833 (48.1)
Missing	79 (0.8)	18 (0.2)
Gestational age at delivery		
< 37 weeks	592 (6.3)	559 (5.6)
37–41 weeks	8713 (92.1)	9434 (93.9)
≥ 42 weeks	9 (0.1)	14 (0.1)
Missing	150 (1.6)	44 (0.4)
HDP		
Yes	388 (4.1)	493 (4.9)
No	8965 (94.7)	9530 (94.8)
Missing	111 (1.2)	28 (0.3)
GDM		
Yes	239 (2.5)	228 (2.3)
No	9114 (96.3)	9795 (97.5)
Missing	111 (1.2)	28 (0.3)

Data show means ± SD or n (%); GDM, gestational diabetes mellitus; HDP, hypertensive disorder in pregnancy; JPA NEO, Axiom Japonica Array NEO; JPAv2, Axiom Japonica Array v2; NVP, nausea and vomiting of pregnancy; SD, standard deviation

### GWAS for association with NVP at the discovery stage

**Fig. 2:**
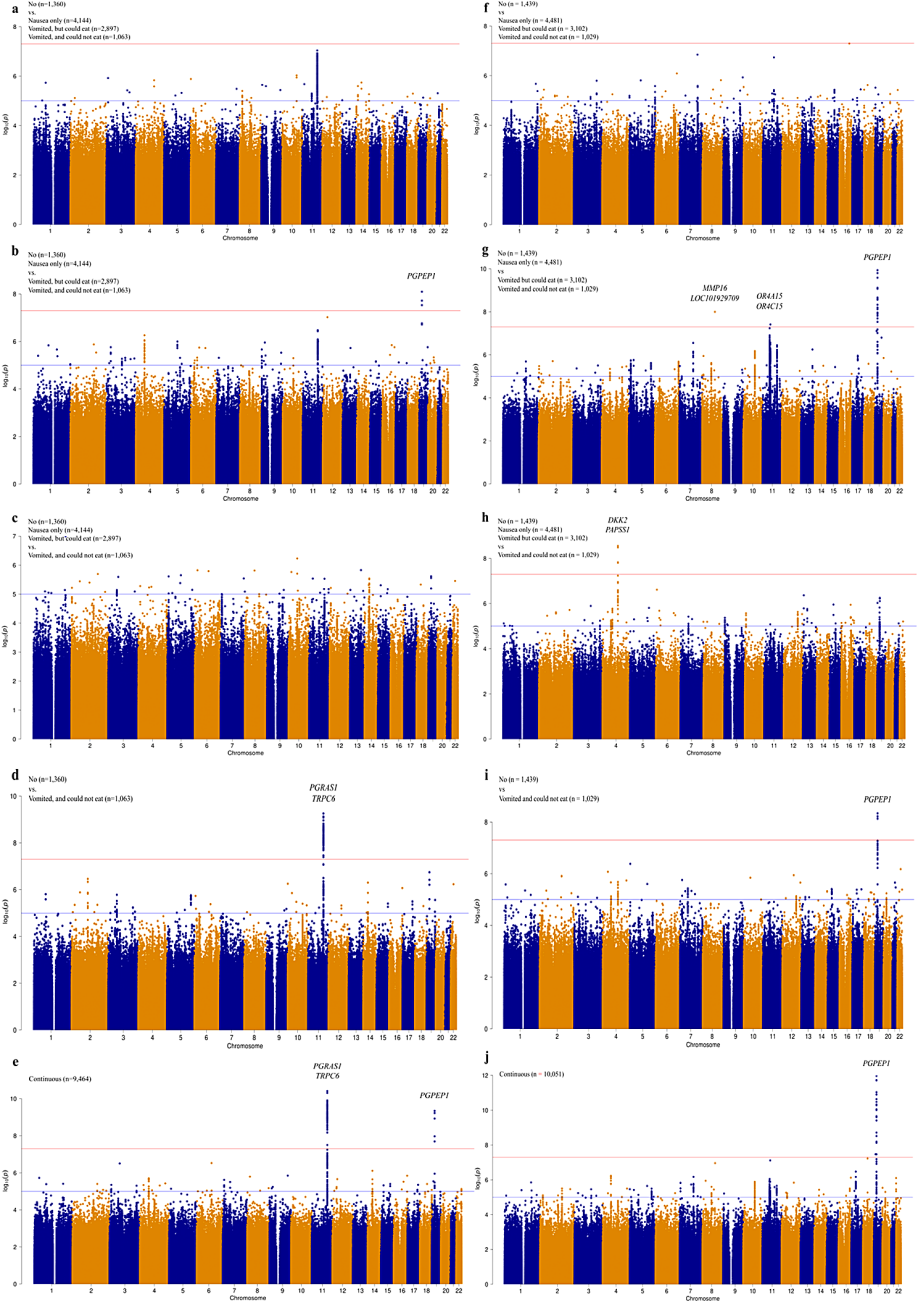
Genome-wide association study (GWAS) for association with NVP. **(a–e)** Manhattan plot of GWAS with the NVP variable as (**a)** binary 1 (“no” vs. “nausea only”, “vomited but could eat,” and “vomited and could not eat”), **(b)** binary 2 (“no” and “nausea only” vs. “vomited but could eat” and “vomited and could not eat”), (**c)** binary 3 (“no”, “nausea only” and “vomited but could eat” vs. “vomited and could not eat”), (**d)** binary 4 (“no” vs. “vomited and could not eat”), and (**e**) continuous (regarding four categories as consecutive severity grading) at the discovery stage. (**f–j**) Manhattan plot of GWAS with the NVP variable as **(f)** binary 1, **(g)** binary 2, **(h)** binary 3, **(i)** binary 4, and **(j)** continuous at the replication stage. Red and blue lines indicate regions that reached genome-wide significance (*p* = 5.0 × 10^− 8^) and suggestive (*p* = 1.0 × 10^− 5^) levels. GWAS, genome-wide association studies; NVP, nausea and vomiting of pregnancy

GWAS was performed to analyze NVP by severity by classifying NVP into the following five patterns: binary 1, binary 2, binary 3, binary 4, and continuous (Fig. 2a–e; Table 2). The locus rs749451 at 19p13.11 reached genome-wide significance levels in binary 2 (Fig. 2b; Table 2). The locus rs77775955 at 11q22.1 was verified to have reached genome-wide significance levels in binary 4 (Fig. 2d; Table 2). In the discovery stage, two loci, rs77775955 at 11q22.1 and rs28568614 at 19p13.11, were found to have reached genome-wide significance levels (Fig. 2e; Table 2). The 11q22.1 region was found between *PGRAS1* and *TRPC6*, wherein 19p13.11 was located in the 3′ untranslated region (UTR) of *PGPEP1*. Both loci have been previously reported in European populations [5]. No significant variants were identified in binaries 1 or 3 (Fig. 2a and c; Table 2).

**Table 2:** Association of NVP with genomic loci meeting significance thresholds at the discovery stage

							Discovery stage (JPA NEO)				Replication stage (JPAv2)				Meta-analysis	
Cytoband	Sentinel SNP	Position (hg19)	Gene	Function	Ref	Alt	AF1	β (SE)	*p*-value		AF1	β (SE)	*p*-value		*p*-value	Direction
Binary 2																
19p13.11	rs749451	19:18479647	*PGPEP1*	UTR3	T	C	0.49	0.184 (0.032)	8.04E-09		0.49	0.202 (0.031)	1.16E-10		5.55E-18	+ +
Binary 4																
11q22.1	rs77775955	11:101247714	*PGR-AS1, TRPC6*	intergenic	G	A	0.56	−0.392 (0.063)	5.54E-10		0.53	−0.182 (0.064)	4.46E-03		1.42E-10	− −
Continuous																
11q22.1	rs77775955	11:101247714	*PGR-AS1, TRPC6*	intergenic	G	A	0.55	−0.090 (0.014)	3.89E-11		0.55	−0.046 (0.013)	4.11E-04		9.75E-13	− −
19p13.11	rs28568614	19:18479019	*PGPEP1*	UTR3	A	G	0.49	0.085 (0.014)	4.54E-10		0.48	0.091 (0.013)	1.87E-12		5.66E-21	+ +

AF1, allele 1 frequency; Alt, alternative allele; β, regression coefficient; JPA NEO, Axiom Japonica Array NEO; JPAv2, Axiom Japonica Array v2; NVP, nausea and vomiting of pregnancy; Ref, reference allele; SE, standard error, SNP, single nucleotide polymorphism; UTR, untranslated region

### GWAS for association with NVP at the replication stage and meta-analysis

**Fig. 3:**
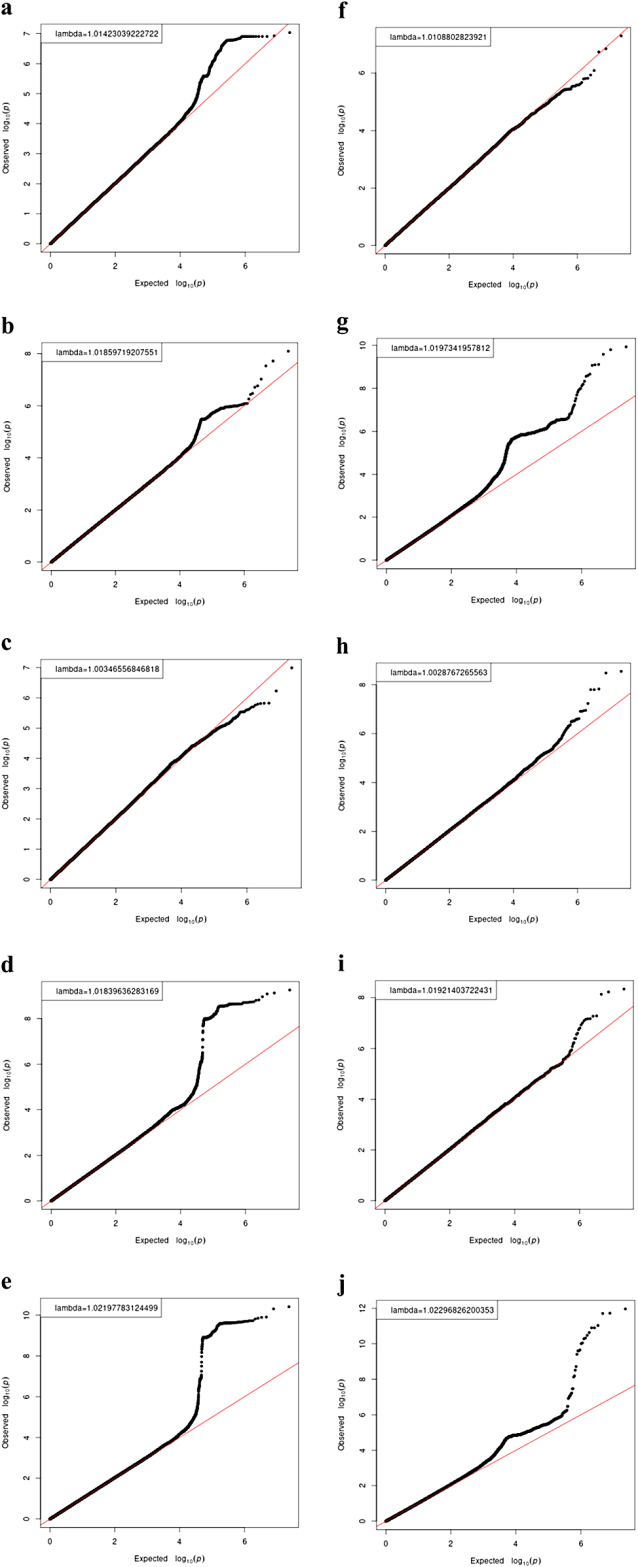
The Q‒Q plot for association with NVP. **(a–e)**The Q‒Q plot with the NVP variable as (**a)** binary 1 (“no” vs. “nausea only”, “vomited but could eat,” and “vomited and could not eat”), **(b)** binary 2 (“no” and “nausea only” vs. “vomited but could eat” and “vomited and could not eat”), (**c)** binary 3 (“no”, “nausea only,” and “vomited but could eat” vs. “vomited and could not eat”), (**d)** binary 4 (“no” vs. “vomited and could not eat”), and (**e**) continuous (regarding four categories as consecutive severity grading) at the discovery stage. (**f–j**) The Q‒Q plot with the NVP variable as **(f)** binary 1, **(g** binary 2, **(h)** binary 3, **(i)** binary 4, and **(j)** continuous at the replication stage. NVP, nausea and vomiting of pregnancy

The participants included in the replication stage were genotyped to validate the two NVP-related gene regions identified in the discovery stage (Table 2). The results showed an association at one of the two gene regions, 19p13.11 (with *p* < 1.0 × 10^− 5^), in both binary 2 and continuous. However, no association with the threshold *p*-value of < 1.0 × 10^− 5^ was confirmed for loci at 11q22.1 in binary 4 and continuous. In the meta-analysis that combined the discovery- and replication-stage results, associations with *p* < 5.0 × 10^− 8^ were confirmed at loci at both gene regions, and the direction of the effect was also consistent. The GWAS results at the replication stage are presented in Fig. 2f–j. In addition to the loci identified in the discovery stage, GWAS in the replication stage identified rs149783780 at 8q21.3 and rs2867907 at 11q11 in binary 2 and rs75393668 at 4q25 in binary 3, which met genome-wide significance levels. Based on the annotations and LocusZoom analysis results, 11q11 was located in a region rich in olfactory receptors, such as *OR4A15* and *OR4C15*, whereas 4q25 was located between *DKK2* and *PAPSS1*. Quantile‒quantile (Q‒Q) plots for all GWAS analyses are shown in Fig. 3. Regional association plots for the discovery and replication stages are shown in Additional file 1, Fig. S1. The results of the functional analysis with HaploReg are shown in Additional file 2, Tables S1–S6. Subsequently, the loci that met genome-wide levels at the discovery or replication stage were examined on the GTEx portal, which revealed that rs28568614, located at 19p13.11, was associated with expression of *LRRC25* in the muscularis of the esophagus. No significant eQTLs were found for the other variants.

## Discussion

The loci rs749451 at 19p13.11 and rs77775955 at 11q22.1 in binary 2 (“no” and “nausea only” vs. “vomited but could eat” and “vomited and could not eat”) and rs28568614 at 19p13.11 in continuous patterns reached the significance threshold in the discovery stage. Of these, the loci at 19p13.11 (rs749451 and rs28568614) reached a significance level of *p* < 1.0 × 10^− 5^ in the replication stage. In addition, in the meta-analysis that integrated the discovery and replication stages results, all three loci at both regions (19p13.11 and 11q22.1) met the significance level of *p* < 5.0 × 10^− 8^. These loci have been reported in a previous study [5]. Although no significant differences were observed in the discovery stage, GWAS in the replication stage identified rs149783780 at 8q21.3 and rs2867907 at 11q11 in binary 2 (“no” and “nausea only” vs. “vomited but could eat” and “vomited and could not eat”) and rs75393668 at 4q25 in binary 3 (“no,” “nausea only,” and “vomited but could eat” vs. “vomited and could not eat”) as NVP-related loci.

The locus rs77775955 at 11q22.1, upstream of *TRPC6*, reached the significance threshold in the discovery stage and meta-analysis, but no significant association was identified in the replication stage. GWAS in a European population showed that a *TRPC6*-related variant, rs2508362 at 11q22.1, was associated with HG [5]. Functional analysis using HaploReg could not identify any variants significantly (with LD values of r^2^ > 0.2) associated with rs77775955 (Additional file 2, Table S1). However, when we analyzed it using Locuszoom, multiple variants with strong associations (LD values of r^2^ > 0.2) were identified (Additional file 1, Fig. S1b and c). *TRPC6* encodes a non-selective cation channel that allows the uptake of essential elements, such as iron [24, 25] and zinc [26, 27], and is expressed at higher levels in the human placenta [28]. Furthermore, a variant near *TRPC6*, rs3018700 at 11q22, was associated with preeclampsia in a GWAS in a Finnish population [29]. The results of the present study suggest that the variation in the *TRPC6*-related variant may affect not only preeclampsia, which occurs mainly in the second trimester of pregnancy but also cause NVP symptoms in early pregnancy. This locus is also located downstream of *PGR-AS1*, a long non-coding RNA that is associated with the progesterone receptor signaling pathway. Previous GWAS have reported an association between loci close to the *PGR* and HG [5, 30].

Variants in the 19p13.11 region, located in the 3ʹ UTR of *PGPEP1*, are in close proximity to *GDF15* (Additional file 1, Fig. S1a, d, e, i, and j). A previous study has shown that variants located in *PGPEP1* are associated with blood GDF15 levels [31]. GDF15 is a member of the TGF-β superfamily and is highly expressed in placental trophoblasts [32]. GDF15 may promote fetal survival by promoting placentation and suppressing the production of inflammatory cytokines to maintain pregnancy [32]. In addition to its role in pregnancy, GDF15 has been shown to regulate body weight and appetite by activating neurons in the hypothalamus and posterior brainstem (vomiting center) [33, 34]. Concordant with our study, a previous GWAS in European populations reported that a *GDF15*-related variant, rs16982345 at 19p13.11, was also associated with HG [5]. Furthermore, in a comprehensive study using whole exome sequencing, one common variant was exome-wide significant in GDF15 and another rare variant in GDF15 that increased the risk of HG by more than 10-fold was identified, suggesting a strong association between *GDF15* and HG [35]. Furthermore, both fetal GDF15 production and maternal susceptibility have also been shown to be associated with HG risk [8]. In addition, the loci identified in this study, rs749451 and rs28568614 at 19p13.11, had an LD value of r^2^ > 0.2 with the locus rs16982345 reported in a previous study (Additional file 2, Table S2 and 3).

In the replication stage GWAS analysis, rs149783780 at 8q21.3, with unknown surrounding function, rs2867907 at 11q11 in the olfactory receptor-rich region in binary 2 (“no” and “nausea only” vs. “vomited but could eat” and “vomited and could not eat”), and rs75393668 at 4q25 in binary 3 reached the significance level (*p* < 5.0 × 10^− 8^; Fig. 2, Table 3). In the 11q11 region, a wide range of variants showed high *p*-values, indicating the presence of numerous olfactory receptors within the region. It is possible that the sequence of this olfactory receptor-rich region, 11q11, causes differences in olfactory sensitivity between individuals, which may influence NVP symptoms. The locus rs75393668 at 4q25 was identified only when the most severe symptom was the case of “ vomited and could not eat,” which suggests that this locus is associated with severe NVP. The candidate gene *DKK2* is an antagonist of catenin signaling and is involved in early embryonic development [36]. However, to our knowledge, the association of *DKK2* with NVP symptoms such as anorexia has not been reported. Therefore, further studies are required to explore the role of these loci in NVP. Moreover, as these three loci were significant only in the GWAS at the replication stage, their validation is essential.

**Table 3:** Association of NVP with genomic loci meeting significance thresholds at the replication stage

							Discovery stage (JPA NEO)				Replication stage (JPAv2)				Meta-analysis	
Cytoband	Sentinel SNP	Position (hg19)	Gene	Function	Ref	Alt	AF1	β (SE)	*p*-value		AF1	β (SE)	*p*-value		*p*-value	Direction
Binary 2																
19p13.11	rs749451	19:18479647	*PGPEP1*	UTR3	T	C	0.49	0.184 (0.032)	8.04E-09		0.49	0.202 (0.031)	1.16E-10		5.546E-18	++
8q21.3	rs149783780	8:90026699	*-*	-	CAAAA	CA	0.01	-0.042 (0.145)	7.73E-01		0.01	0.833 (0.145)	1.00E-08		8.95E-05	-+
11q11	rs2867907	11:55170876	*OR4A15, OR4C15*	Intergenic	C	T	0.50	-0.017 (0.033)	6.09E-01		0.50	-0.178 (0.032)	3.87E-08		1.73.E-05	--
Binary 3																
4q25	rs75393668	4:108157443	*DKK2, PAPSS1*	Intergenic	T	C	0.91	0.121 (0.088)	1.69E-01		0.91	-0.542 (0.091)	2.80.E-09		8.96E-04	+-
Binary 4																
19p13.11	rs749451	19:18479647	*PGPEP1*	UTR3	T	C	0.50	0.312 (0.063)	6.17E-07		0.49	0.373 (0.064)	4.61E-09		1.80E-14	++
Continuous																
19p13.11	rs749451	19:18479647	*PGPEP1*	UTR3	T	C	0.49	0.084 (0.014)	5.97E-10		0.49	0.092 (0.013)	1.08E-12		4.42E-21	++

AF1, allele 1 frequency; Alt, alternative allele; β, regression coefficient; JPA NEO, Axiom Japonica Array NEO; JPAv2, Axiom Japonica Array v2; NVP, nausea and vomiting of pregnancy; Ref, reference allele; SE, standard error, SNP, single nucleotide polymorphism; UTR, untranslated region

This study had several limitations. First, the NVP variable used in this study was self-reported and collected through a questionnaire rather than a medical diagnosis. However, the rate of NVP among participants (approximately 85%) detected in this study is close to that reported in a previous study (80%) [1]. Additionally, the NVP-related questions to define the four patterns (binary 1–4) in this study were straightforward and clear; thus, misclassification was likely to be low. Second, a functional analysis of model organisms was not performed for the loci related to NVP identified in this study. Third, the statistical power was low because of the small sample size of the GWAS. Fourth, selection bias may exist, and the extrapolation of findings may be limited because all participants were Japanese. However, one of the strengths of this study is that it is based on a prospective birth cohort design, the TMM BirThree Cohort Study and replication was performed on separate participants within the same cohort.

In conclusion, this study in the Japanese population successfully identified different NVP-related loci at 11q22.1 and 19p13.11, as reported in previous studies in a European population. The findings of this study provide further insights into the generalizability of previous GWAS on the association between genetic background and NVP. Collectively, these findings can assist in developing therapies targeting relevant proteins at identified loci and new methods for prediction and diagnosis.

### Electronic supplementary material

Below is the link to the electronic supplementary material.

Supplementary Material 1

Supplementary Material 2

## Data Availability

The datasets used and/or analysed during the current study are available from the corresponding author on reasonable request.

## Abbreviations

AF: Allele frequency
eQTL: Expression quantitative trait loci
fastGWA: Fast MLM-based genome-wide association tool
fastGWA-GLMM: Fast GLMM-based genome-wide association tool
GTEx: Genotype-tissue expression
GWAS: Genome-wide association study
HG: Hyperemesis gravidarum
HWE: Hardy–Weinberg equilibrium
IDB: Identity by descent
JPA NEO: Axiom Japonica Array NEO
JPAv2: Axiom Japonica Array v2
LD: Linkage disequilibrium
MAF: Minor allele frequency
NVP: Nausea and vomiting of pregnancy
QC: Quality control
Q‒Q: Quantile‒quantile
SD: Standard deviation
SE: Standard error
TMM: BirThree Cohort Study Tohoku Medical Megabank Project Birth and Three Generations Cohort Study
