# Supplementary material for: Genome-wide association study of nausea and vomiting during pregnancy in Japan: the TMM BirThree Cohort Study
Source: BMC Pregnancy Childbirth. 2024 Mar 20;24:209. doi: 10.1186/s12884-024-06376-4 (PMC10953086; doi:10.1186/s12884-024-06376-4)
Supplement: Supplementary file 1 — Supplementary Material 1 [file 12884_2024_6376_MOESM1_ESM.pptx]

## Slide 1
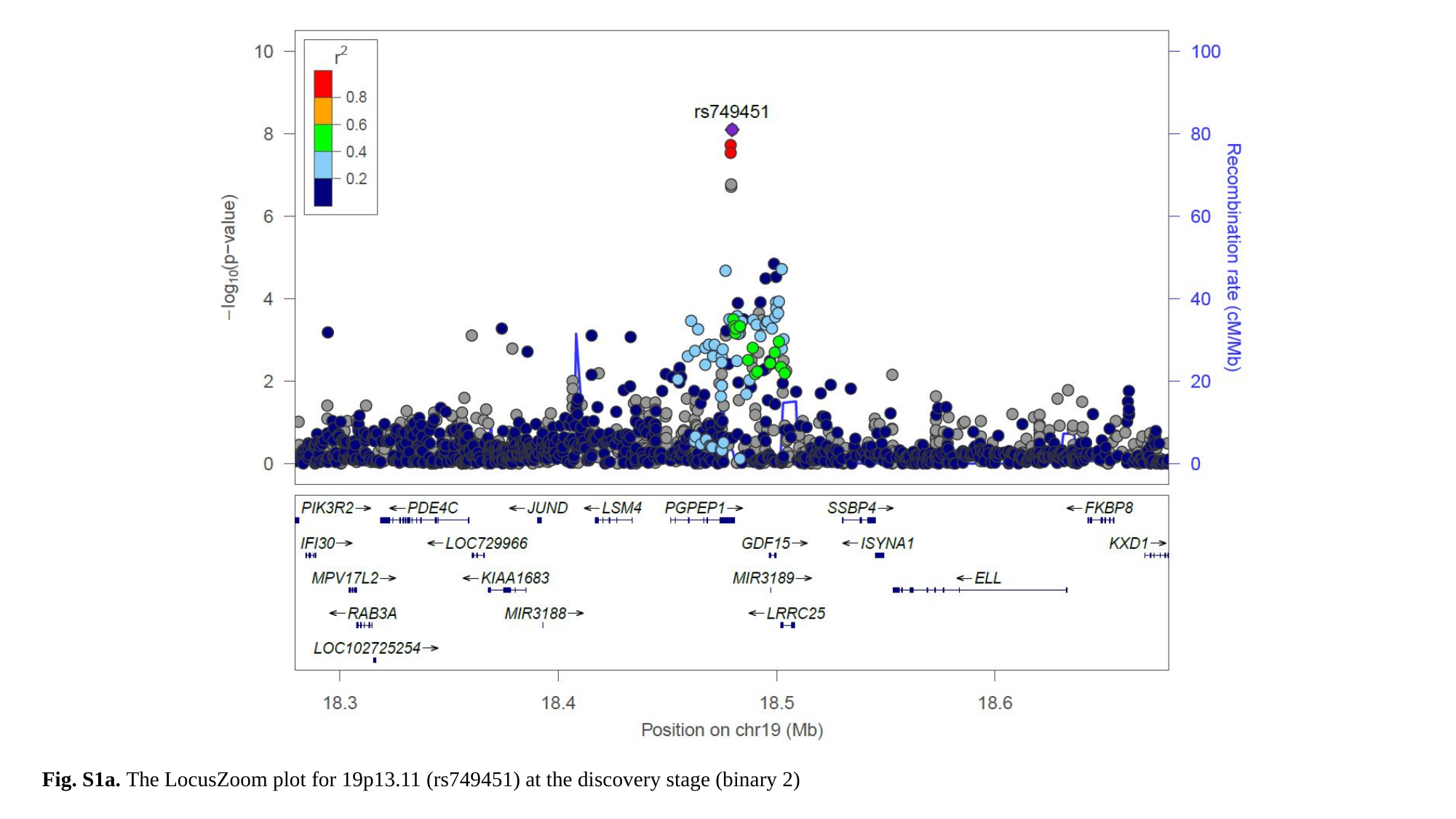

Fig. S1a. The LocusZoom plot for 19p13.11 (rs749451) at the discovery stage (binary 2)

## Slide 2
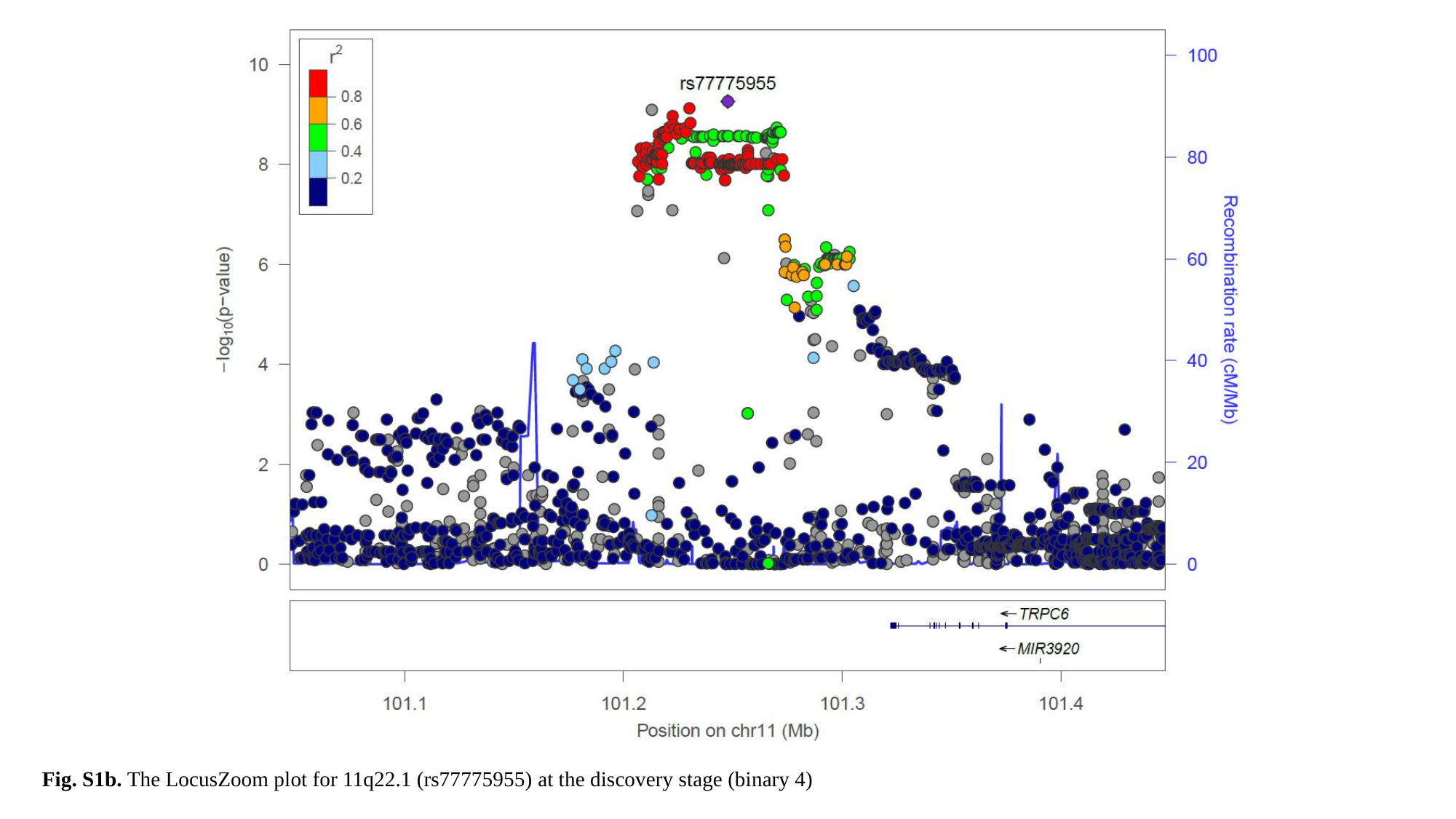

Fig. S1b. The LocusZoom plot for 11q22.1 (rs77775955) at the discovery stage (binary 4)

## Slide 3
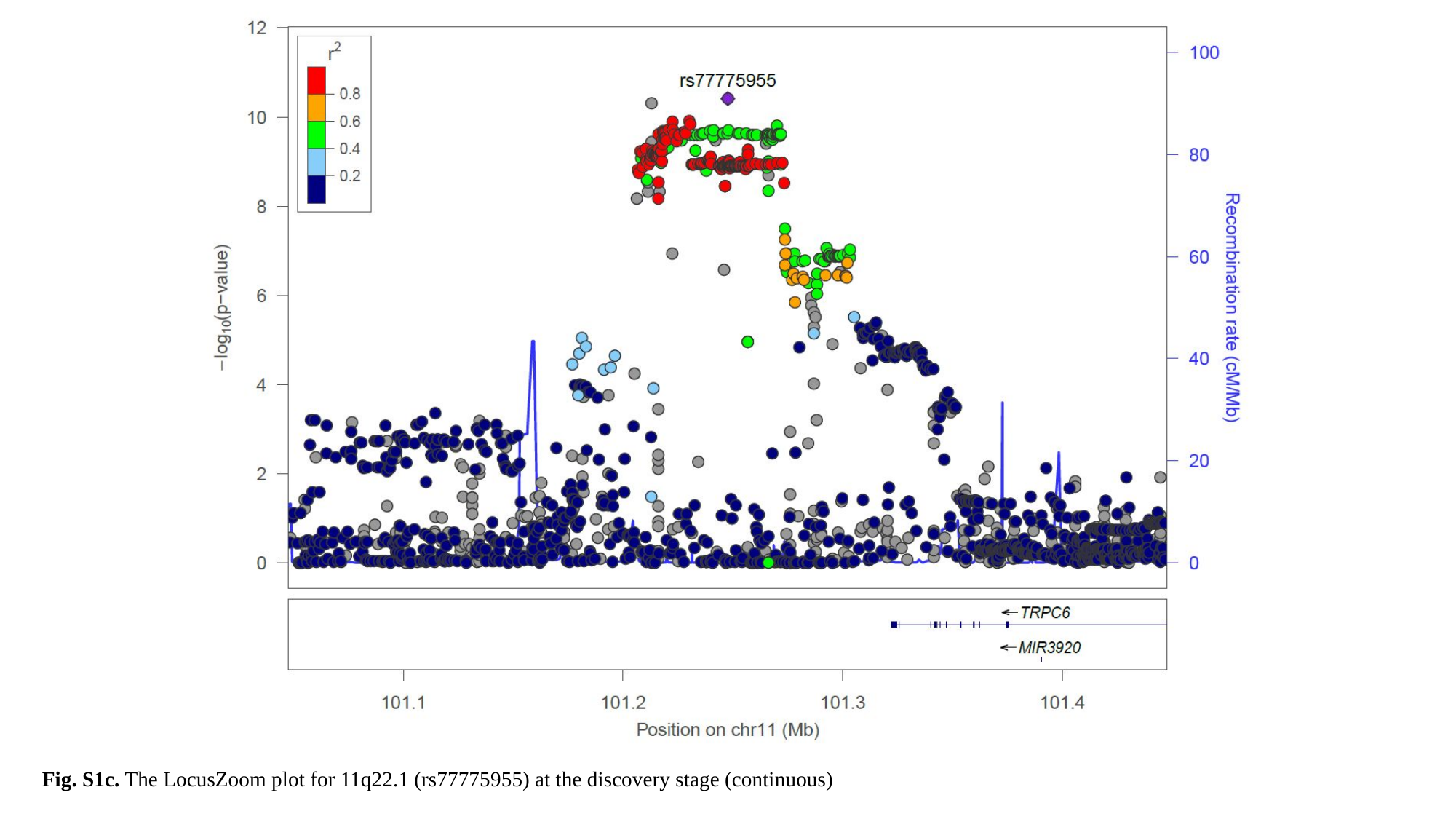

Fig. S1c. The LocusZoom plot for 11q22.1 (rs77775955) at the discovery stage (continuous)

## Slide 4
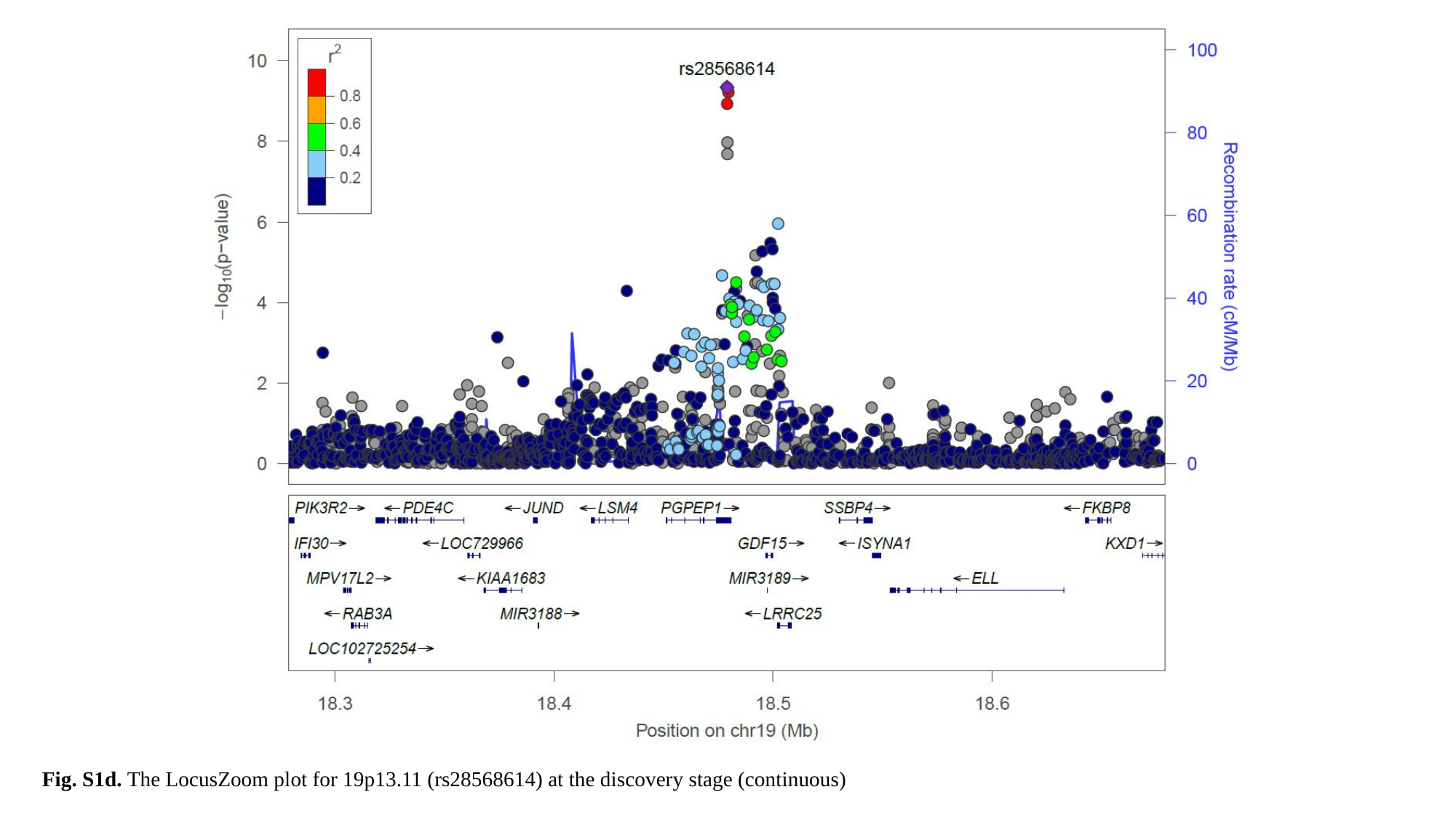

Fig. S1d. The LocusZoom plot for 19p13.11 (rs28568614) at the discovery stage (continuous)

## Slide 5
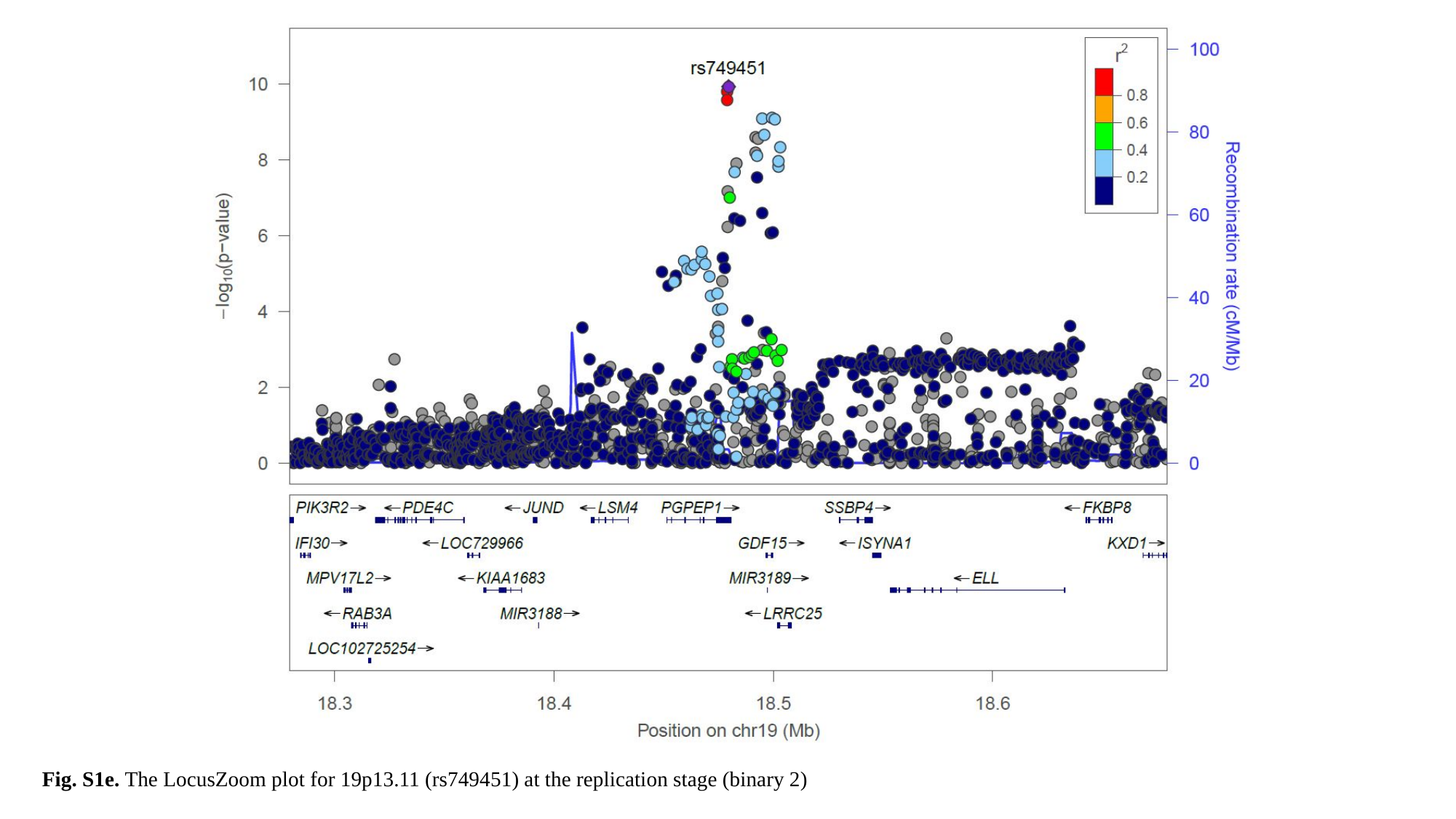

Fig. S1e. The LocusZoom plot for 19p13.11 (rs749451) at the replication stage (binary 2)

## Slide 6
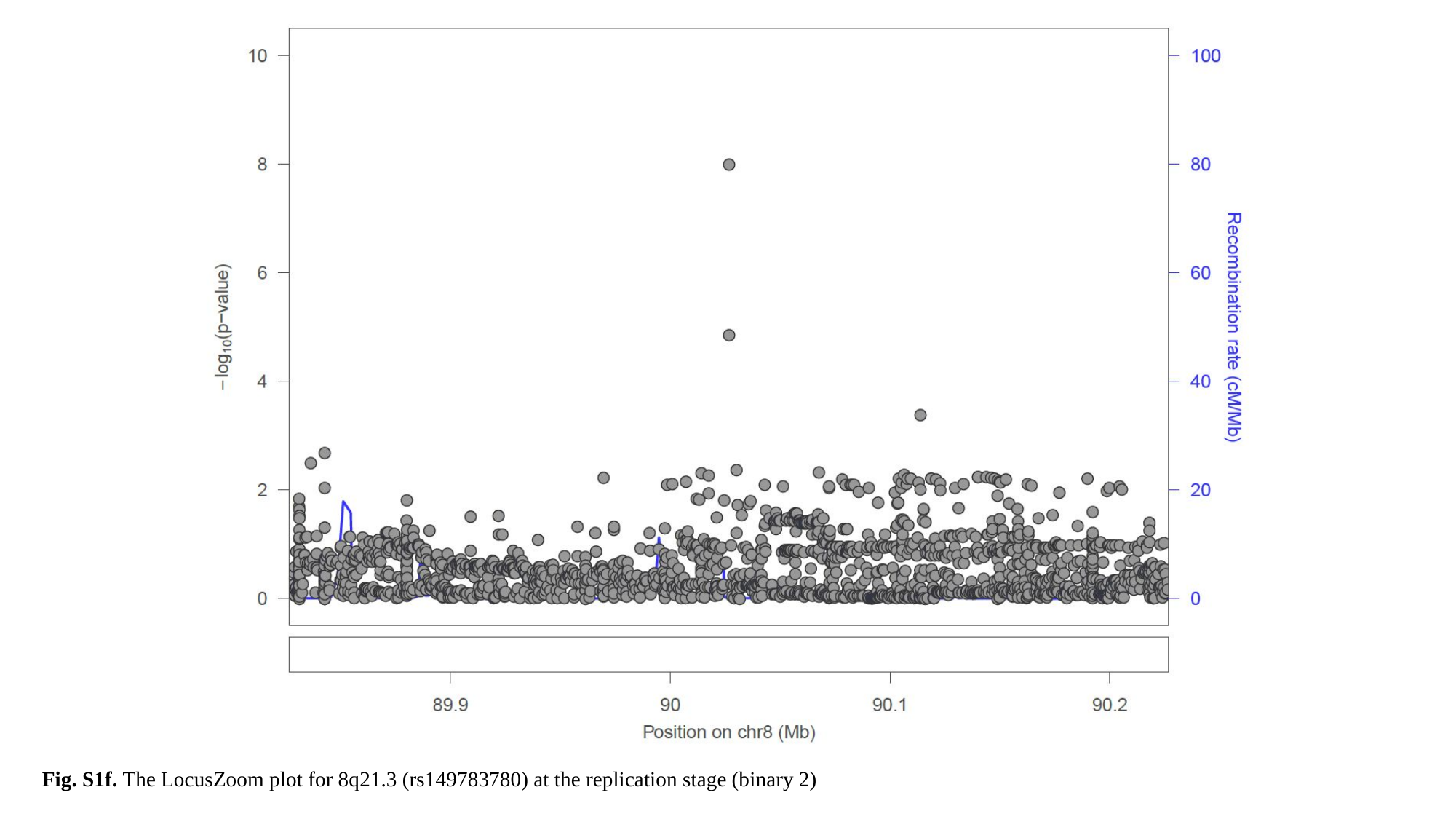

Fig. S1f. The LocusZoom plot for 8q21.3 (rs149783780) at the replication stage (binary 2)

## Slide 7
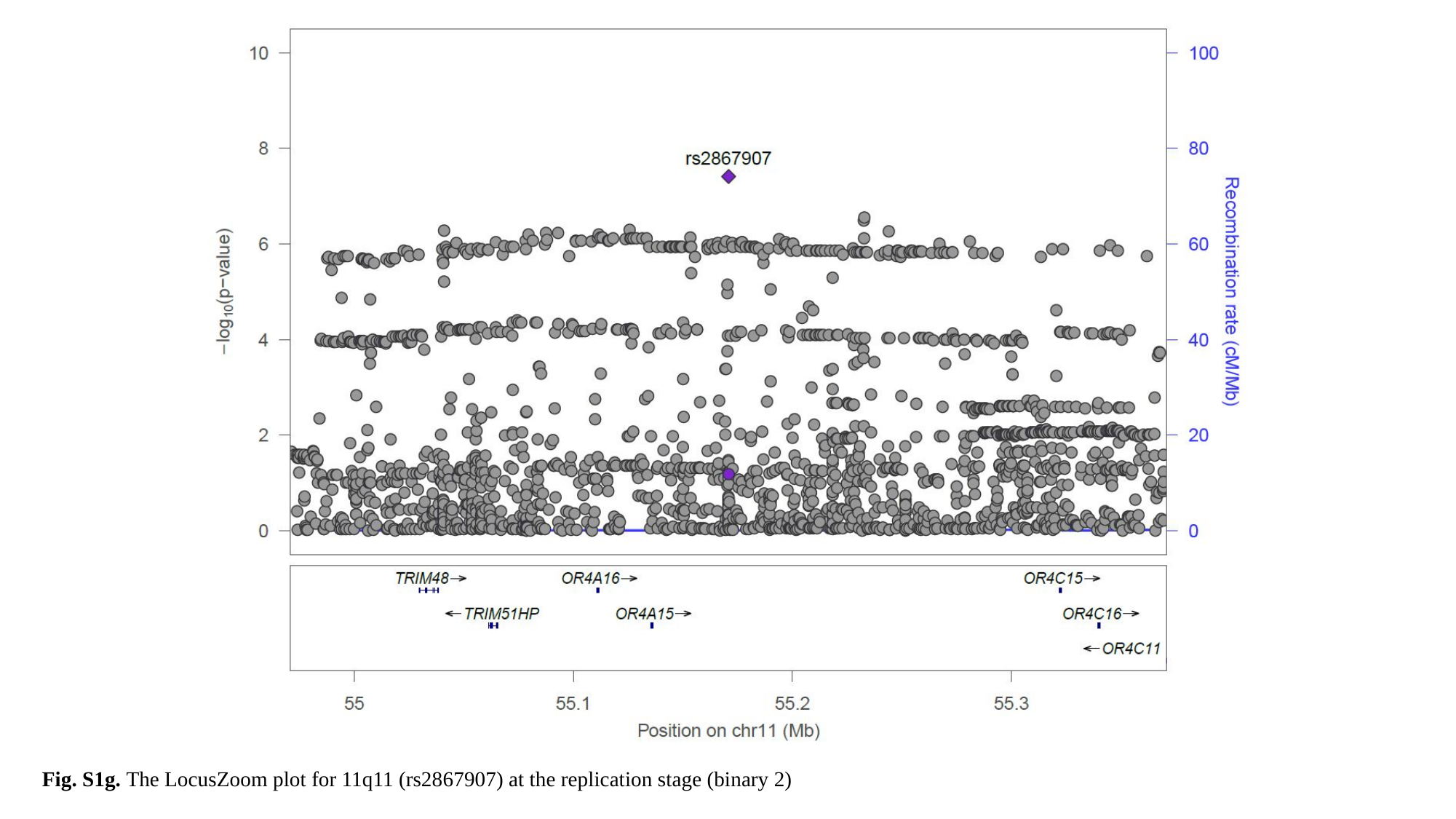

Fig. S1g. The LocusZoom plot for 11q11 (rs2867907) at the replication stage (binary 2)

## Slide 8
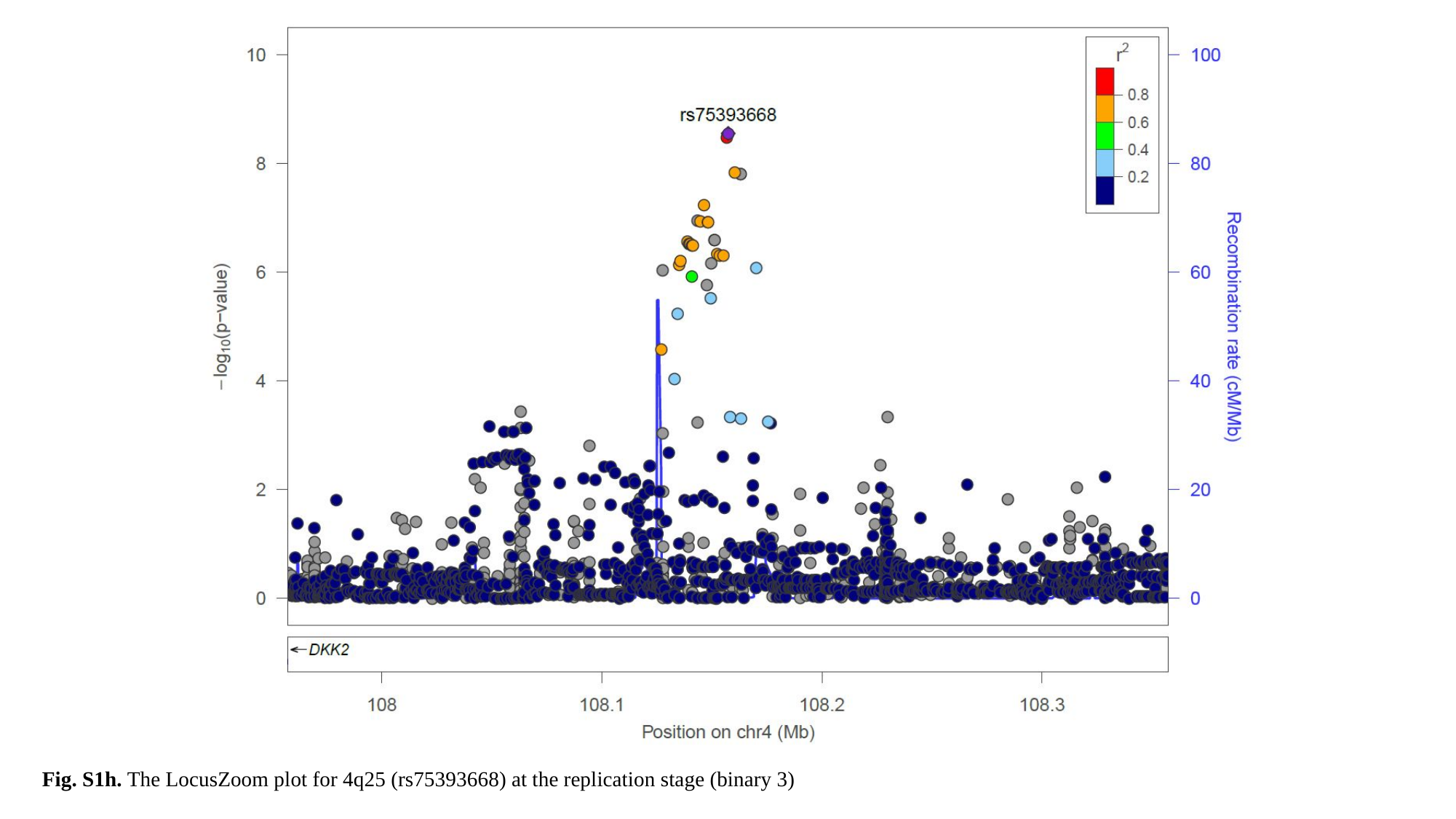

Fig. S1h. The LocusZoom plot for 4q25 (rs75393668) at the replication stage (binary 3)

## Slide 9
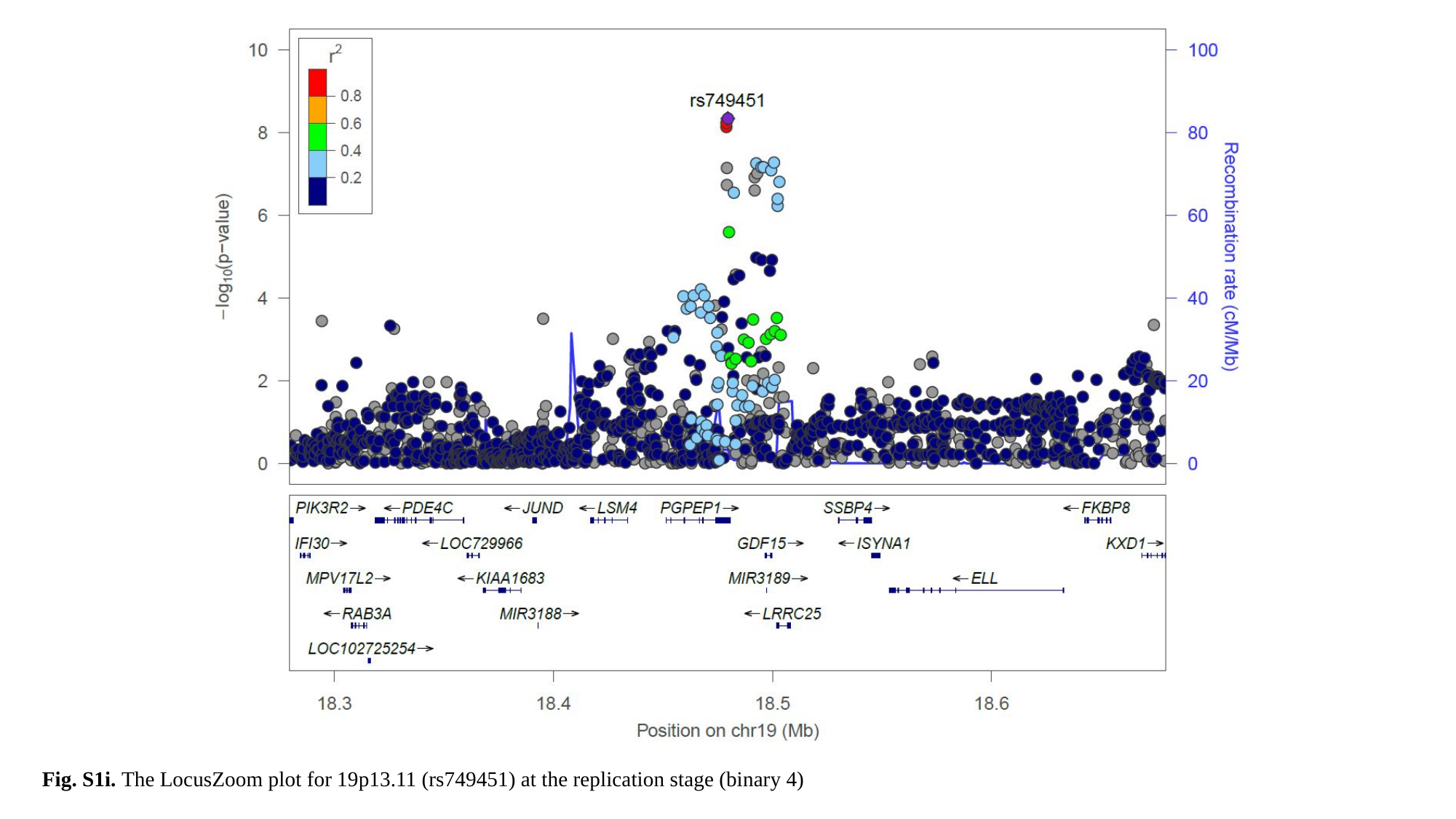

Fig. S1i. The LocusZoom plot for 19p13.11 (rs749451) at the replication stage (binary 4)

## Slide 10
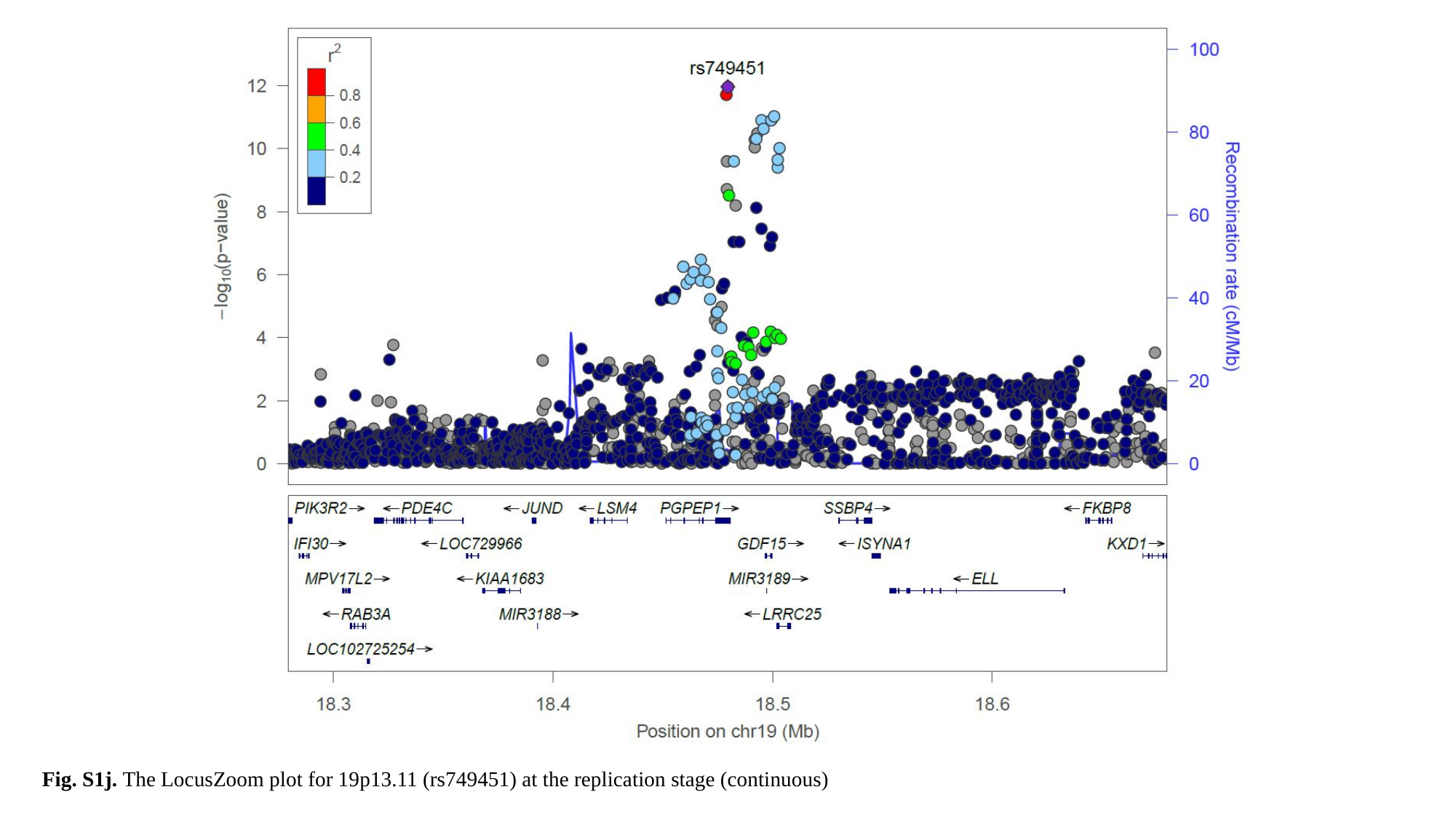

Fig. S1j. The LocusZoom plot for 19p13.11 (rs749451) at the replication stage (continuous)
